# Selection, characterization, and biosensing applications of DNA aptamers targeting cyanotoxin BMAA†

**DOI:** 10.1039/d4ra02384f

**Published:** 2024-04-26

**Authors:** Xaimara Santiago-Maldonado, José A. Rodríguez-Martínez, Luis López, Lisandro Cunci, Marvin Bayro, Eduardo Nicolau

**Affiliations:** a Department of Chemistry, University of Puerto Rico San Juan PR 00925-2437 USA eduardo.nicolau@upr.edu xaimara.santiago@upr.edu luis.lopez14@upr.edu lisandro.cunci@upr.edu marvin.bayro@upr.edu +1-787-522-2150 +1-787-292-9820; b Department of Biology, University of Puerto Rico San Juan PR 00925-2437 USA jose.rodriguez233@upr.edu; c Molecular Science Research Center, University of Puerto Rico San Juan 00931-3346 USA

## Abstract

Scientists have established a connection between environmental exposure to toxins like β-*N*-methylamino-l-alanine (BMAA) and a heightened risk of neurodegenerative disorders. BMAA is a byproduct from certain strains of cyanobacteria that are present in ecosystems worldwide and is renowned for its bioaccumulation and biomagnification in seafood. The sensitivity, selectivity, and reproducibility of the current analytical techniques are insufficient to support efforts regarding food safety and environment monitoring adequately. This work outlines the *in vitro* selection of BMAA-specific DNA aptamers *via* the systematic evolution of ligands through exponential enrichment (SELEX). Screening and characterization of the full-length aptamers was achieved using the SYBR Green (SG) fluorescence displacement assay. Aptamers BMAA_159 and BMAA_165 showed the highest binding affinities, with dissociation constants (*K*_d_) of 2.2 ± 0.1 μM and 0.32 ± 0.02 μM, respectively. After truncation, the binding affinity was confirmed using a BMAA-conjugated fluorescence assay. The *K*_d_ values for BMAA_159_min and BMAA_165_min were 6 ± 1 μM and 0.63 ± 0.02 μM, respectively. Alterations in the amino proton region studied using solution nuclear magnetic resonance (NMR) provided further evidence of aptamer–target binding. Additionally, circular dichroism (CD) spectroscopy revealed that BMAA_165_min forms hybrid G-quadruplex (G4) structures. Finally, BMAA_165_min was used in the development of an electrochemical aptamer-based (EAB) sensor that accomplished sensitive and selective detection of BMAA with a limit of detection (LOD) of 1.13 ± 0.02 pM.

## Abstract 2

Scientists have established a connection between environmental exposure to toxins like β-*N*-methylamino-l-alanine (BMAA) and a heightened risk of neurodegenerative disorders.

## Graphical abstract

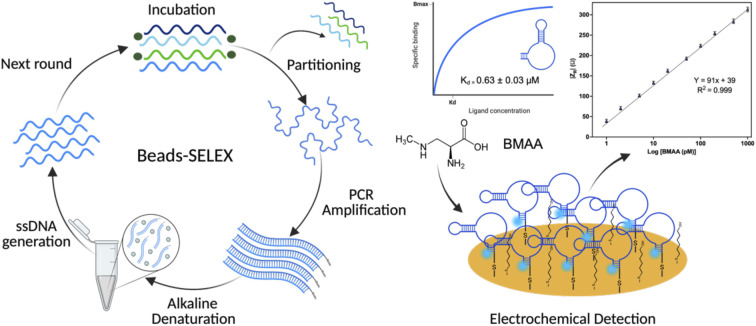

## Introduction

In recent years, cyanobacterial blooms have increased in freshwater and marine ecosystems as a result of global warming and human-induced eutrophication.^1^ Cyanobacteria are known to produce toxic secondary metabolites with diverse mechanisms of action that pose a threat to aquatic ecosystems and human health.^2,3^ BMAA is a noncanonical amino acid frequently produced by cyanobacteria and other microorganisms in marine, freshwater, brackish water, and terrestrial ecosystems.^4,5^ It was first identified over 50 years ago on the island of Guam, where the prevalence of neurodegenerative disorders was 50–100 times greater than the global average.^4,5^ Later research linked the consumption of BMAA-contaminated foods, such as cycad seed flour and flying foxes, to the Guamanian Amyotrophic Lateral Sclerosis/Parkinsonism Dementia Complex (ALS/PDCO).^6–8^ Numerous investigations have been conducted since then to understand its role in pathological processes of neurodegeneration.^9–13^ BMAA's capacity to act as an agonist for various glutamate receptors and its misincorporation into proteins have been proposed as the two primary mechanisms underlying its neurotoxic effects.^14–19^ BMAA has been detected in ecosystems throughout the world at different trophic levels, and it is known to bioaccumulate in common foods such as fish, mussels, lobsters, and crabs.^2^ Currently, ingestion of contaminated seafood and water and inhalation of aerosolized toxins are the main routes of BMAA exposure in humans.^20,21^

The detection and quantification of BMAA in biological and environmental samples remains a challenging task.^2,22^ In contaminated water sources, BMAA can occur as a free amino acid at concentrations in the parts-per-trillion (ng L^−1^) range.^23^ It can also be found bound to proteins, forming metal complexes, and organic colloids, among others.^2,22,24^ Its structural isomers, *N*-(2-aminoethyl) glycine (AEG), β-amino-*N*-methylalanine (BAMA) and 2,4-diamonibutyric acid (DAB) often coexist in the same environments, with BAMA also exhibiting neurotoxic effects.^25^ The majority of analytical techniques for quantifying BMAA use either liquid or gas chromatography coupled with mass spectrometry (MS) or tandem mass spectrometry (MS/MS) detectors. Alternative non-chromatographic methods such as capillary electrophoresis (CE), ^1^H nuclear magnetic resonance, and enzyme-linked immunosorbent assays (ELISA) have also been explored. Yet, only ultra-performance liquid chromatography (UPLC-MS/MS) using pre-column 6-aminoquinoyl-*N*-hydroxysuccinimidyl carbamate (AQC) derivatization has been validated according to the Association of Official Analytical Chemists (AOAC).^2^ Large disparities in the outcomes of comparable samples examined by different analytical methods have sparked debates over BMAA's widespread presence in a variety of environments.^2,22,26,27^ Ineffective sample preparation along with the limited selectivity, sensitivity, and reproducibility of the available techniques hinder the advancement of public safety initiatives.^2^ Because of this, it is crucial to develop novel analytical methods for the reliable detection of BMAA in diverse sample matrices.

Given BMAA's small size, polarity, and structural resemblance to other small compounds, an analytical technique combining high sensitivity and selectivity is required. Aptamers are examples of molecular recognition elements (MREs) that can provide the necessary analyte specificity for bioanalytical applications.^28,29^ These are short single-stranded synthetic oligonucleotides identified through the exponential enrichment method known as systematic evolution of ligands (SELEX).^30,31^ The formation of three-dimensional structures, like stem-loops, bulges, and/or hairpins, creates binding pockets for target interaction.^28,32^ The ability to distinguish between closely related molecules, like isomers, is made possible by the specificity of these interactions.^33^ Compared to conventional MREs like antibodies, aptamers are not limited to immunogenic targets and have several benefits, including lower molecular weight, greater sensitivity, selectivity, and stability.^28,34–36^ They also have a lower production cost and can regain functionality after several regeneration steps.^28,37,38^ Moreover, aptamers can be used in conjunction with innovative signal transduction technologies to produce highly sensitive aptamer-based sensors for the detection of low-abundance analytes. Over the last decade, studies have described the identification of aptamers against various cyanotoxins and their successful applications in biosensing.^39,40^ However, the selection of BMAA-specific aptamers has yet to be addressed.

Herein, the identification of the DNA aptamers against cyanotoxin BMAA is presented for the first time. The *in vitro* selection of the aptamers and characterization of their binding affinity are described. The integration of the selected aptamer in a biosensing platform for the electrochemical detection of BMAA is also presented.

## Experimental

### Materials

All DNA oligos and DNA library Prep MC UNI Kit with full-length indexed adapters (xGen UDI-UMI adapters) were obtained from Integrated DNA Technologies (Coralville, IA, USA) (Tables S1 & S2†). The SELEX library was designed by the Sooter laboratory at West Virginia University (WVU).^41^ Biotinylated BMAA was synthesized by Pepscan (Lelystad, Netherlands). Dynabeads™ M-280 Streptavidin was acquired from Invitrogen (Carlsbad, CA, USA). Econotag PLUS 2X Master Mix (pH 9.0, 0.1 units per μL of EconoTaq DNA polymerase, 400 μM dNTPs, 3 mM MgCl_2_) was from Lucigen (Middleton, WI, USA). Polymerase chain reaction (PCR) purification was achieved using a QIAquick PCR Purification Kit from Qiagen (Germantown, MD, USA). DNA purification from agarose gels was performed with the QIAquick Gel Extraction Kit from Qiagen (Germantown, MD, USA). Lambda Exonuclease was purchased from New England Biolabs (NEB; Ipswich, MA). Sodium chloride, magnesium chloride, 1 M Tris–HCl (pH = 8), streptavidin-coated agarose resin, glycogen, molecular-grade ethanol, mono-butyl phthalate, atenolol, *S*(+)-2-amino-3-(methylamino) propionic acid hydrochloride (l-BMAA, ≥97% NMR), aminoethylglycine (AEG), 2,4-aminobutyric acid (DAB), SYBR® Green I nucleic acid gel stain (10 000× in DMSO), Multiscreen® 96-well plates (flat bottom clear and black), Nuclease-Free Water (Molecular Biology Grade), Tris(2-carboxyethyl)phosphine hydrochloride (TCEP), potassium hexacyanoferrate(iii), potassium hexacyanoferrate(ii), Methylene Blue (Atto MB2 NHS ester), and 6-mercapto-1-hexanol were purchased directly from Sigma Aldrich (St. Louis, MO, USA). Invitrogen™ SYBR™ Gold Nucleic Acid Gel Stain (10 000× concentrate in DMSO), Pierce™ Streptavidin Coated Immunoassay Plates (96-well black) were purchased from Thermo Fisher Scientific (Waltham, MA, USA). Flow-through columns were from Bio-Rad Laboratories, Inc. (Hercules, CA, USA). Gold (Au) electrode 3.0 mm diameter, coiled platinum wire auxiliary electrode (23 cm), and RE-5B Ag/AgCl (3 M NaCl) reference electrode were purchased from Bioanalytical Systems, Inc. (West Lafayette, IN, USA). Nanopore water (18.2 MΩ cm^2^, Milli-Q Direct 16) was always used unless otherwise stated.

### *In vitro* selection of aptamers

**Fig. 1:**
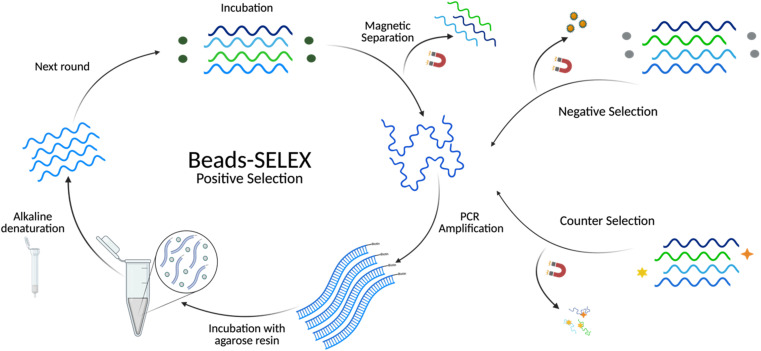
Schematic representation of the *in vitro* selection using Bead-SELEX. During positive selection rounds, BMAA (green spheres) was incubated with the library pool. For the negative selection, the immobilization matrix with no BMAA (gray spheres) was incubated with the library pool. The counter selection involved the incubation of the library with structurally similar compounds (yellow star, orange diamond).

A selection buffer (SB) composed of 100 mM NaCl, 5 mM MgCl_2,_ and 20 mM Tris (pH 7.6) was used throughout the selection. Before each selection round, aptamers were heated to 90 °C for 5 min and cooled at room temperature for 15 min. All binding reactions had a total volume of 500 μL and were incubated with rotation at room temperature. Biotinylated BMAA (Fig. S1B†) was dissolved in SB to a concentration of 50 μM and immobilized onto streptavidin magnetic beads, according to the manufacturer's directions, to form the immobilization target (IT). For the first round of positive selection (R1), the IT was incubated with 2000 pmoles of ssDNA library at equal molar ratios for 48 h. Following incubation, unbound ssDNAs were removed by magnetic separation, washed 3 times with SB, and resuspended in 200 μL of SB. Subsequent selection rounds were performed using 200 pmoles of ssDNA pool and incubated with equal molar ratios of the target. The same process was followed for positive selections through round 5 with decreasing incubation times. For the first round of negative selection (R4), the biotinylated linker (no target) was immobilized onto streptavidin magnetic beads, forming the immobilization substrate (IS). This was incubated with the ssDNA pool for 1 h. Following incubation, magnetic separation was used to recover the supernatant with the unbound sequences. Supernatants from 3 consecutive washes with SB were also recovered to serve as a template for amplification. The same process was followed for the remaining negative selections with increasing incubation times. Competitive elution (CE) rounds (R6–R11) consisted of an incubation of IT followed by magnetic separation and resuspension in a free BMAA (Fig. S1A†) solution. As in the negative selections, the supernatant was collected to serve as a template for amplification. The target concentration and incubation times were decreased each round. Finally, negative selection rounds from R10 to R16 consisted of an incubation with IT followed by incubation with structurally similar targets (Fig. S2†). Then, the beads were washed and resuspended in SB to be used as templates for amplification. Detailed selection conditions are provided in Table S3† and a schematic representation of the process detailed above is represented in Fig. 1.

PCR reactions were carried out using a T100 Thermal Cycler from Bio-Rad Laboratories, Inc. (Hercules, CA) with a primer concentration of 400 nM. Thermal cycling conditions were 95 °C for 5 min [95 °C for 1 min, 6 °C for 45 s, 72 °C for 1 min] and a final extension of 72 °C for 7 min. For every selection round, low-volume (50 μL) PCR reactions were carried out with varying numbers of cycles and analyzed in 4% agarose gels to determine the optimal number of PCR cycles. Then, large-scale PCR reactions (4 mL) were performed using the determined number of cycles and purified using the QIAquick PCR Purification Kit. The concentration of dsDNA was measured with NanoDrop One/One Microvolume UV-Vis Spectrophotometer from (Thermo Fisher, Waltham, MA, USA). Products were analyzed in 4% agarose gels stained with ethidium bromide (120 V for 40 min). Gel images were obtained using the Azure Sapphire Biomolecular Imager (Azure Biosystems, Inc., Dublin, CA, USA). When required, purifications from 2% agarose gels were achieved by cutting the band of interest with a razor and extracting dsDNA following the QIAquick Gel Extraction Kit protocol.

For the generation of ssDNA, streptavidin agarose resin was incubated with dsDNA (100 μL of resin per 50 μg of dsDNA) for 2 h at room temperature with rotation. After incubation, the mixture was transferred to a flow-through column and washed with 2.5 mL of PBS 1×. Then, ssDNA was eluted with 2 mL of a 20 mM NaOH solution in triplicate (using the same flow-through). The ssDNA was ethanol precipitated using 10 mg mL^−1^ of glycogen, 0.1 volumes of ammonium acetate (3 M, pH 5.2), and 3 volumes of ethanol at −80 °C for 1 h. Afterward, the samples were centrifuged at 4 °C for 60 minutes, dried, and resuspended in SB. The ssDNA concentration was determined using the NanoDrop and the product was analyzed on a denaturing urea PAGE (8% polyacrylamide 7 M urea, 1.0 mm, 90 V for 1.5 h), followed by staining with SYBR Gold for at least 30 min. Gel images were obtained using the Azure Sapphire Biomolecular Imager. If necessary, gel purification was carried out following the standard crush and soak method. Briefly, the desired band was excised from the gel, crushed, and extracted overnight with a buffer containing 10 mM magnesium acetate tetrahydrate, 0.5 M ammonium acetate, 1 mM EDTA (pH 8.0) at 30 °C, followed by ethanol precipitation as previously described.

### Sequencing and analysis

Ten samples were sent for high-throughput sequencing (HTS). Two samples corresponded to the original library. From SELEX, rounds 3, 8, 9, 12, 13, 16, 17, and 18 were selected. The libraries corresponding to each round were amplified using PCR. The purified products were used as DNA templates for the library preparation method. End-repair and adapter ligation were carried out per manufacturer instructions using full-length adapter sequences with unique dual indexes (UDIs). After ligation, the fully indexed products were purified using a QIAquick PCR Purification Kit, pooled into one sample, and purified from 2% agarose gel. The dsDNA concentration was measured with a Qubit 4 Fluorometer using a 1× dsDNA HS (high sensitivity) assay kit (Invitrogen, Carlsbad, CA, USA). An aliquot of 50 μL from the PCR sample was sent for high-throughput sequencing using the Illumina HiSeq PE150 system (Novogene Corporation Inc., Sacramento, CA, USA).

Cutadapt^42^ and Trimmomatic^43^ were used as trimming tools for HTS data pre-processing. The first analysis of HTS data was achieved using the AptaSUITE^44^ pipeline *via* the graphical user interface (GUI). Base distribution analysis across the selection and preliminary analysis of the sequence distributions and enrichment were achieved with this platform. Within AptaSUITE, the AptaTRACE algorithm identified possible sequence-structure binding motifs (k-mer size of 6) for both Beads-SELEX and GO-SELEX. The GUI of the FASTAptamerR 2.0 (ref. 45) pipeline was used for sequence counts, sequence enrichment, clustering, motif analysis across populations, data filtration, and plotting. Enrichment is defined as the normalized values (reads per million) of the last round of selection (R18) divided by the normalized values of the library control (R0). Secondary structures, free energies (Δ*G*_s_), and melting temperatures (*T*_m_) were predicted using the Mfold^3^ software.

### SG fluorescence displacement assay

The SYBR Green (SG) assay was modified from previous studies.^46–48^ SG (2×) and target dilutions were prepared in SB. Aptamers were heated to 90 °C for 5 min, then 4 μL of aptamer (10 μM) were mixed with 4 μL SG 2× and incubated for 30 min at room temperature with rotation. Then, 8 μL of the mixture was transferred to a black 96-well plate, with wells containing 117 μL varying concentrations of BMAA (0, 0.049, 0.098, 0.20, 0.39, 0.78, 1.6, 3.1, 6.3, 13, 25 μM), for a total volume of 125 μL. Control samples included SG 2×, target, and aptamer alone, as well as target–SG and target–aptamer mixtures. Fluorescence was measured in an Infinite 200 PRO microplate reader (Tecan, Männedorf, Switzerland) using an excitation wavelength of 480 nm and an emission wavelength of 520 nm. Fluorescence at 520 nm was used to calculate the signal response:1
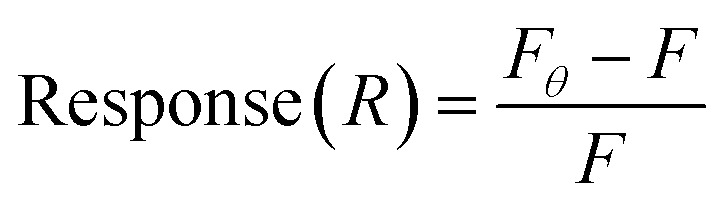
where *F*_*θ*_ is the fluorescence without a target and *F* is at a given concentration of the target. The dissociation constant (*K*_d_) was determined through nonlinear regression analysis by fitting the data with one site-specific binding equation. Experiments were performed in triplicate. Cross-reactivity assays were made similarly but using a fixed analyte concentration of 12.5 μM.

### BMAA-conjugated fluorescence assay

This assay was modified from a previously described affinity chromatography method.^38,47,49^ Streptavidin-coated 96-well plates were used to conjugate biotinylated BMAA. Aptamer and target dilutions were prepared in SB. Before the experiment, aptamers were heated to 90 °C for 5 min and cooled for 15 min at room temperature. First, each well was washed with 200 μL of SB 3 times. Then, 100 μL of 10 μM biotinylated BMAA was added to each well and incubated for 2 h at room temperature. The wells were washed with 200 μL of SB 3 times. Aliquots of 100 μL of FAM-labeled aptamer solutions at varying concentrations (0, 0.08, 0.16, 0.32, 0.63, 1.3, 2.5, 5.0, 10, 20 μM) were added to the wells and incubated for 30 min at room temperature with shaking. Finally, the wells were washed with 200 μL of SB 3 times and resuspended in 100 μL of SB. For the negative controls, 1 μM FAM-labeled aptamers were added to previously washed wells (no target) and incubated for 30 min at room temperature. Fluorescence was measured in an Infinite 200 PRO microplate reader with an excitation wavelength of 480 nm and emission at 520 nm. Fluorescence response was calculated using eqn (2). The dissociation constant (*K*_d_) was determined through nonlinear regression analysis by fitting the data with one site-specific binding equation. All samples were prepared in tricates.2
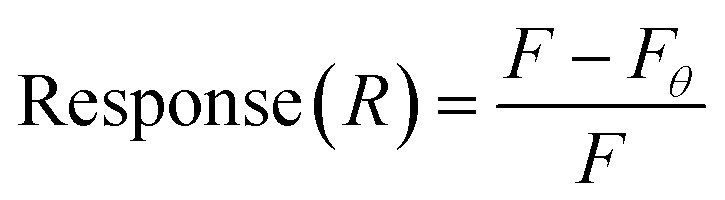


### Nuclear magnetic resonance

Solution NMR spectra were recorded on a Bruker AVANCE III HD spectrometer operating at a ^1^H Larmor frequency of 500 mHz a Prodigy BBO Cryoprobe (Bruker, Billerica, MA, USA). One-dimensional excitation sculpting ^1^H spectra were acquired at 298 K with a 2 second recycle delay, 45.3 μs dwell time, and a 1.49 ms acquisition period. All samples were dissolved in 20 mM sodium phosphate buffer with 100 mM NaCl and 2 mM MgCl_2_. All NMR samples included 10% D_2_O. Before NMR analysis, aptamers were heated to 90 °C for 5 min and cooled for 15 min at room temperature. For the titration experiments, a series of spectra were recorded upon the addition of aliquots containing molar equivalents in steps of 0.0625 : 1, up to 3 : 1 ligand : aptamer, according to the concentration of each aptamer (20 μM BMAA_159 and 14.4 μM BMAA_165). Each spectrum was the average of 2048 transients. Changes in frequency intensity at the amino proton region (7.93 ppm for BMAA_159 and 7.83 ppm for BMAA_165) were plotted against the molar fraction of the target to create binding isotherms.

### Circular dichroism spectroscopy

Samples consisting of 10 μM BMAA, 10 μM aptamers (full-length and minimers), and aptamer/target mixtures (1 : 1 molar ratio, 30 min incubation at room temperature) were prepared in SB. Circular dichroism (CD) spectra were recorded on a Jasco J-810 CD spectropolarimeter (Jasco, Inc., Easton, MD) from 320 to 210 nm in a 1 mm path-length quartz cuvette at room temperature. Data gathered were the average of 5 scans collected in units of millidegrees (mdeg) *versus* wavelength at a scanning rate of 100 nm min^−1^. Scan of SB buffer alone was subtracted from the average scans of all samples.

### Electrochemical detection of BMAA

All electrochemical measurements were done using a three-electrode electrochemical cell with an Au electrode (bare or aptamer-modified) as the working electrode, a platinum wire as the counter electrode, and an Ag/AgCl reference electrode. A Reference 600+ Gamry potentiostat was used for the electrochemical measurements, and the Gamry Instruments Framework software was used for data collection. The cyclic voltammetry (CV) experiments were performed by scanning between −0.2 and 0.6 V at a scan rate of 50 mV s^−1^. For EIS, an amplitude of 10 mV, an initial frequency of 5 MHz, and a final frequency of 10 Hz were used. SWV was measured in a potential window between −0.4 and 0 V with a pulse size of 25 mV and a frequency of 25 Hz. Before modification, Au electrodes were polished in silica slurry of various particle sizes (1.0, 0.3, and 0.05 μm) and washed with nanopure water. The electrodes were electrochemically cleaned in 0.1 M H_2_SO_4_ using CV by scanning between 0 and 1.6 V for 250 cycles, then rinsed with nanopure water. For aptamer immobilization, 2 μL of 100 μM aptamer and 40 μL of 5 mM TCEP were incubated for 1 h. Clean Au electrodes were incubated with 10 μL of the aptamer/TCEP mixture overnight, then rinsed with nanopure water. Electrochemical characterization of electrodes before and after aptamer modification was carried out in 5 mM [Fe(CN)_6_]^3−/4−^ redox couple solution (1 : 1 molar ratio) in 0.1 M KCl using CV and EIS.

**Fig. 2:**
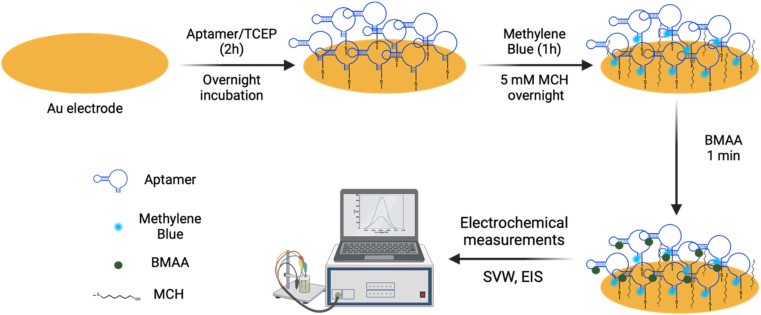
Schematic representation of the EAB sensor fabrication. Aptamer BMAA_165_min was immobilized on Au electrodes through SAMs. Then, MB was covalently linked to the aptamers *via* chemical cross-linking, and the electrode surface was blocked with MCH. Electrochemical detection of BMAA was measured using SWV and EIS.

The aptamer-modified Au electrodes were incubated with 10 μL 1 mM MB solution for 1 h to link MB to aptamers covalently. Afterward, electrodes were rinsed with nanopure water and incubated with 10 μL of 5 mM 6-mercaptoetanol-1-hexanol 9 (MCH) solution overnight. A buffer composed of 4 mM NaCl, 0.2 mM MgCl_2_, and 0.8 mM Tris at pH 7.4 was used for all biosensing experiments and solutions. A 1 μM BMAA master solution was freshly prepared before each experiment and used to prepare standard solutions of 100, 10, and 1 nM for titration into the electrochemical cell. The buffer volume in the electrochemical cell was 15 mL. The detection of BMAA was attained by adding increasing aliquots of target to achieve concentrations between 1 and 1000 pM. Then, the electrochemical cell was stirred for 1 min without disturbing the electrodes. This was followed by a waiting period of 30 s to be certain the solution was stagnant. Electrochemical measurements were performed using SWV and EIS. Cross-reactivity experiments were carried out following the same procedure but using AEG, DAB, or atenolol as analytes. The resistive impedance (*Z*_R_) was used to investigate the biosensor's analytical response using EIS. For this, the EIS spectra of the blank (no analyte) were subtracted from all samples using Gamry Echem Analyst 2 software. Calibration curves were fitted using standard linear regression. The limit of detection (LOD) and limit of quantification (LOQ) were calculated using eqn (3) and (4), respectively, where *S* is the slope of the calibration curve and *σ* is the standard deviation (SD). Fig. 2 provides a schematic representation of EAB sensor fabrication and the electrochemical detection of BMAA.3
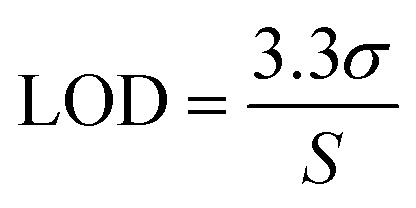
4
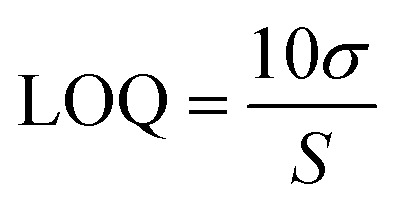


### Statistics

GraphPad Prism 10.2.0 was used for the preparation of all graphs and statistical analysis.

## Results & discussion

### *In vitro* selection

The Beads-SELEX approach was adapted from the previous work of Williams, R. M. *et al.* and Hong, K. L. *et al.*^50,51^ Magnetic beads were used as a solid support to facilitate the separation of target-bound sequences. Negative selection, counter selection, and competitive elution rounds were included at various stages of selection to account for immobilization-related limiting factors like steric hindrance and nonspecific binding.^41,52^ The library input and incubation times were gradually decreased during positive selection rounds to increase the stringency of selection while minimizing the loss of low-frequency high-affinity oligonucleotides. Negative selection rounds were introduced to minimize non-specific binders to the immobilization matrix. The incubation times for the negative selection were continually increased during SELEX to intensify the rigorousness of selection. Competitive elution rounds were added to isolate sequences with a higher affinity towards the free target than the immobilized target. To further promote the enrichment of highly specific aptamers, counter selection rounds were used to select against structurally similar compounds (Fig. S2†). These counter targets included small molecules atenolol and mono-butyl phthalate, both of which fall under the category of contaminants of emerging concern.^53,54^ Likewise, as BMAA and its isomers frequently coexist in heterogeneous environments, AEG and DAB were added in subsequent counter selection rounds.^25^

### Sequencing analysis

**Fig. 3:**
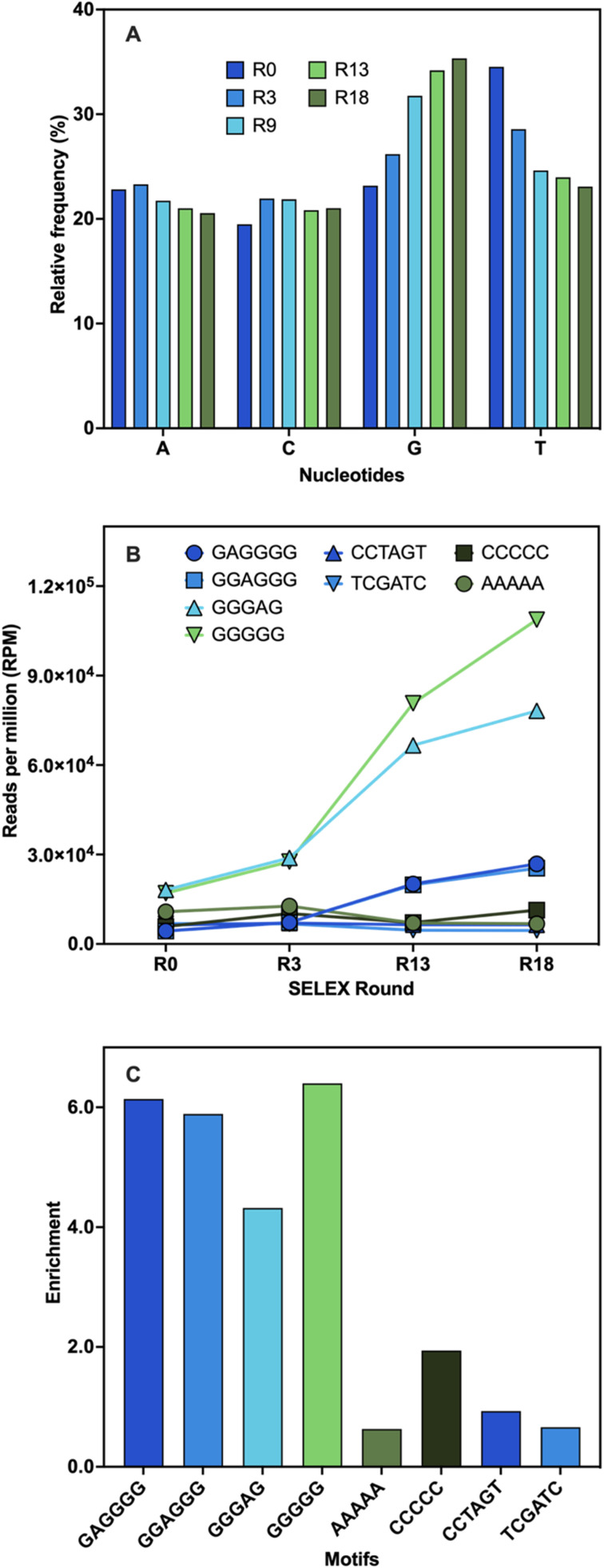
Sequencing analysis. (A) Base distribution analysis of the relative frequency (%) of each nucleotide across different rounds of SELEX compared to the library control (R0). (B) Abundance (RPM) of the motifs predicted by AptaTRACE (GAGGGG, GGAGGG, GGGAG, GGGGG) and the random sequences chosen for comparison (AAAAA, CCCCC, CCTAGT, TCGATC). (C) Enrichment values of the motifs defined as the abundance in R18 divided by the abundance in R0.

The identification of aptamer candidates is essential to the success of any SELEX process. Thousands or millions of sequences in each library can be elucidated through high-throughput sequencing (HTS), making it more suitable for large SELEX library pools than the standard Sanger sequencing.^55^ Many factors can result in high-abundance sequences that might not possess the highest binding capabilities, including PCR bias, non-uniform sequence distribution, energy-driven selection, and non-specific binding.^44,56–59^ Since the best binders may be underrepresented in the pool, selecting sequences based on their maximal abundance is not always ideal.^57,58,60^ Using a variety of bioinformatic techniques, HTS analysis was carried out at various stages of selection to study changes in the library pools. First, the pool composition from several selection cycles were examined and compared to the original library. The base composition of the initial library was 23% A, 19% C, 23% G, and 35% T (Fig. 3A). During SELEX, a 2% frequency decrease in the A content was observed while the C content increased by 2%. The G content showed a 12% frequency increase from the start to the end of the selection along with a 12% decrease in the T content (Fig. 3A). These shifts in base composition suggest the evolution of G-rich sequences during SELEX.

Subsequently, the library pools were examined to identify binding motifs. These are short sequences typically responsible for target binding, and they are often the ones conserved and enriched throughout selection.^55,61^ Once identified, binding motifs can help reduce the pool diversity for further stages of analysis.^55^ Four overrepresented 6-mer motifs were identified: TGGGGG, GGGGAG, GAGGGG, and GGAGGG (Table S4†). To validate these findings, the motifs' frequencies were monitored during several stages of the selection process and their total enrichment was determined. Every motif displayed an increase in abundance that peaked between R3 and R13 and continued to rise until the end of selection (Fig. 3B). This was likely due to the increasing stringency of conditions with the addition of negative and counter selection rounds. A variety of random motifs were included for comparison to ensure that the findings are a direct outcome of the selection process. The enrichment values of all identified motifs were higher than those of the random motifs (Fig. 3C). These results support the findings obtained from the population analysis and establish a connection between the enrichment of sequences with conserved G-rich motifs and the notable changes in base distribution throughout SELEX. High G-content is a common feature in several DNA aptamers, including other small-molecule binding DNA aptamers, and is often associated with the potential to adopt G-quadruplex (G4) structures.^46,60,62^ The enrichment of these motifs might indicate that G-rich sequences are preferred for BMAA binding.

To reduce the pool diversity and narrow down possible candidates, the sequences were clustered. Representative sequences were chosen from each cluster and their secondary structures were predicted. A total of five aptamer candidates were chosen to screen for binders based on consensus families. The sequences of these candidates are summarized in Table S2.†

### Affinity evaluation and characterization of aptamers

Given there is no universal characterization method suitable for all aptamers, various techniques should be employed to confirm aptamer–target binding.^47^ The SG fluorescence displacement assay enables accurate determination of the dissociation constant (*K*_d_) and rapid screening of binders.^46,47^ When SG intercalates in the hybridized regions of the aptamers, fluorescence is substantially increased, and aptamer–target interactions cause detectable changes in fluorescence intensity.^46,47^ This assay was used for screening the selected full-length aptamer candidates. Control experiments showed that fluorescence enhancement was only observed in the presence of aptamers (Fig. S3†). Changes in fluorescence were measured following the addition of various BMAA concentrations. Four candidates displayed BMAA binding, with affinities under 20 μM (Fig. S3†). Candidates BMAA_159 and BMAA_165 were selected for additional characterization due to their lower *K*_d_ values. In both cases, fluorescence decreased as target concentration increased, suggesting that SG was displaced as a result of target binding (Fig. 3A).^46^ Fitting of the binding curves using one site-specific binding showed affinities of 2.2 ± 0.1 μM for BMAA_159 and 0.32 ± 0.02 μM for BMAA_165. A higher binding affinity was observed for BMAA_165. Notably, the aptamers BMAA_159 and BMAA_165 contained the motifs GAGGGG and GGAGGG, respectively, and have similar secondary structures (Fig. S4†). The random region of BMAA_165 was scrambled to provide a control ssDNA sequence (Table S2†). The lack of response supports the involvement of these motifs and the resulting secondary structures in BMAA binding (Fig. 3A). The motifs may interact directly with BMAA or stabilize the bases directly engaged in BMAA binding.^63^ Additionally, the SG assay was used to study the cross-reactivity of the aptamers against potentially interfering compounds. When exposed to BMAA, both aptamers exhibited a considerably higher fluorescence response than the other compounds tested (Fig. 3B). This shows a high degree of selectivity and highlights the significance of negative selection rounds involving possibly interfering substances during the selection process. In summary, aptamer characterization with the SG assay demonstrated that both BMAA_159 and BMAA_165 exhibit high target specificity and selectivity.

**Fig. 4:**
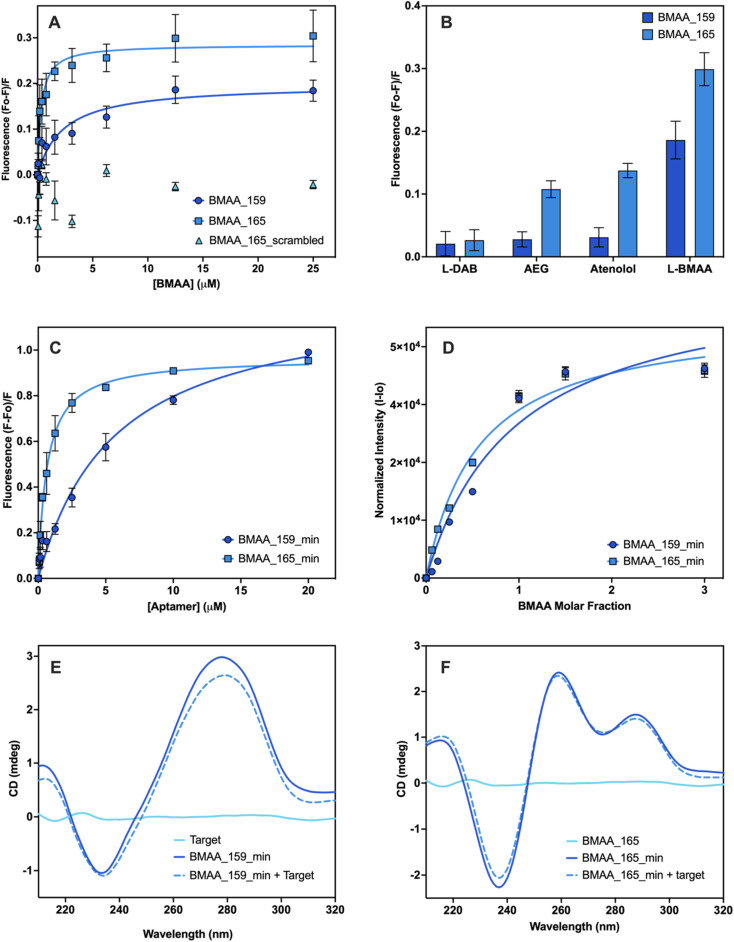
Affinity evaluation and characterization of aptamers. Evaluation of the binding affinity of full-length aptamers using the SG fluorescence displacement. (A) Binding isotherms of BMAA_159 (*K*_d_ = 2.2 ± 0.1), BMAA_165 (*K*_d_ = 0.32 ± 0.02) and BMAA_165_scrambled (*K*_d_ = no binding). (B) Comparison of the fluorescence response of BMAA and structurally related compounds (AEG, DAB, atenolol). A full analysis of the independent experiments can be found in the ESI (Fig. S7, Table S6†). Evaluation of the binding affinity of truncated aptamers using BMAA-conjugated fluorescence assay. (C) Binding isotherms of BMAA_159_min (*K*_d_ = 6 ± 1) and BMAA_165_min (*K*_d_ = 0.63 ± 0.02). A full analysis of the independent experiments can be found in the ESI (Fig. S8, Table S7†). Measurements were performed in triplicates and the error bars represent the calculated standard error. The *K*_d_ values were determined through non-linear regression analysis by fitting the data with one site-specific binding equation using GraphPad Prism 10.2.0. Qualitative affinity evaluation of the truncated aptamers using the frequency intensity changes at the amino proton region measured with solution NMR. (D) Binding isotherms of BMAA_159_min and BMAA_165_min. The amino proton region of the ^1^H NMR spectra is provided in the ESI (Fig. S6†). Spectrums are the average of 2048 transients and error bars represent the signal/noise figure for each measurement. CD spectra of BMAA (10 μM), aptamers (5 μM), and aptamers incubated with BMAA (1 : 2 ratio) recorded in buffer (100 mM KCl, 5 mM MgCl_2,_ and 20 mM Tris, pH of 7.6) for (E) BMAA_159_min and (F) BMAA_165_min. Each CD spectra is the average of 5 scans.

Aptamers BMAA_159 and BMAA_165 were truncated into 40-base long structures (Table S2†) with secondary structures characterized by two stem loops (Fig. S4†). Advantages of truncated aptamers include higher production yield, lower cost, and occasionally, lower *K*_d_ values.^33,64^ An alternative characterization method was explored to evaluate binding affinity. For this assay, BMAA was conjugated on streptavidin-coated 96-well plates and exposed to varying concentrations of FAM-labeled aptamers. It resembles the standard affinity chromatography technique as it involves target immobilization on a solid support.^33,47,64^ An increase in fluorescence intensity was observed with increasing aptamer concentrations (Fig. 4C). The apparent *K*_d_ values for BMAA_159_min and BMAA_165_min were 6 ± 1 μM and 0.63 ± 0.02 μM, respectively. Variations in affinity measurements are to be expected when employing different characterization techniques due to intrinsic differences among methods.^47^ Particularly, the higher *K*_d_ values obtained with this assay can be a result of a decreased affinity of the aptamers towards the immobilized target. It is widely recognized that non-specific binding can occur in affinity measurements where the target is immobilized.^46,47^ The fluorescence response with and without BMAA was examined to assess non-specific binding. When BMAA was present, the fluorescence response was higher for both aptamers (Fig. S5†). The difference in fluorescence response was substantially higher for BMAA_165_min when compared to BMAA_159_min. According to these findings, BMAA_165_min exhibits a lower degree of non-specific adsorption in addition to a higher binding affinity.

Solution NMR was used to confirm further the truncated aptamers' binding affinity along with obtaining structural and dynamic details about the aptamer–target systems.^65^ The amino proton region of the NMR spectra was used for the analysis since the ^1^H NMR signals arising from the nitrogen bases are sensitive to conformational changes and hydrogen bonding interactions.^66^ Each aptamer showed characteristic chemical shift perturbations upon incubation with BMAA. For aptamer BMAA_159_min, the most significant chemical shift perturbations are observed at 7.12 ppm and 7.93 ppm (Fig. S6A†). For BMAA_165_min, the most significant chemical shift perturbations are observed at 7.62 and 7.83 ppm (Fig. S6B†). When plotted, the changes in chemical shift follow a concentration-dependent behavior (Fig. 4D). This phenomenon points to the formation of new hydrogen bonds or conformational changes during BMAA binding.^66^ Aptamer BMAA_165_min achieved saturation at lower BMAA concentrations, which correlates with a higher affinity towards the target. The results are consistent with the BMAA binding patterns seen in previous studies.

CD spectroscopy was used to investigate the secondary structures of the truncated aptamers with and without BMAA. The CD spectrum of BMAA_159_min (Fig. 4E) showed a negative band at 240 nm and a positive band at 275 nm, characteristic of B-form DNA structures.^41,67^ A reduction in the CD amplitude was observed after incubation with BMAA. Prior research has linked this amplitude reduction to a transition from a B-form duplex to a hairpin.^48,67^ These stem-loop structures are frequently linked to target binding and additional stabilization to the secondary structure.^48^ The CD spectrum of BMAA_165_min showed a negative peak at 235 nm and positive peaks at 260 nm and 290 nm (Fig. 4F). These peaks are consistent with the formation of hybrid G4 structures (parallel and antiparallel).^68–70^ Following BMAA incubation, no significant changes in ellipticity were observed, suggesting that the hybrid G4 conformation was maintained. G-rich oligomers capable of folding into G4 structures comprise a large group of aptamers with several advantages over other unstructured sequences.^62,71^ G4 structures have demonstrated greater resistance against certain nucleases and are more chemically and thermodynamically stable.^71^ Because of their larger negative charge density, they also have better electrostatic interactions with positively charged ligands.^71^ Based on the characterization results, BMAA_165_min was identified as the most promising aptamer candidate due to its superior binding affinity, selectivity, and capacity to form highly stable G4 structures.

### Electrochemical detection of BMAA

**Fig. 5:**
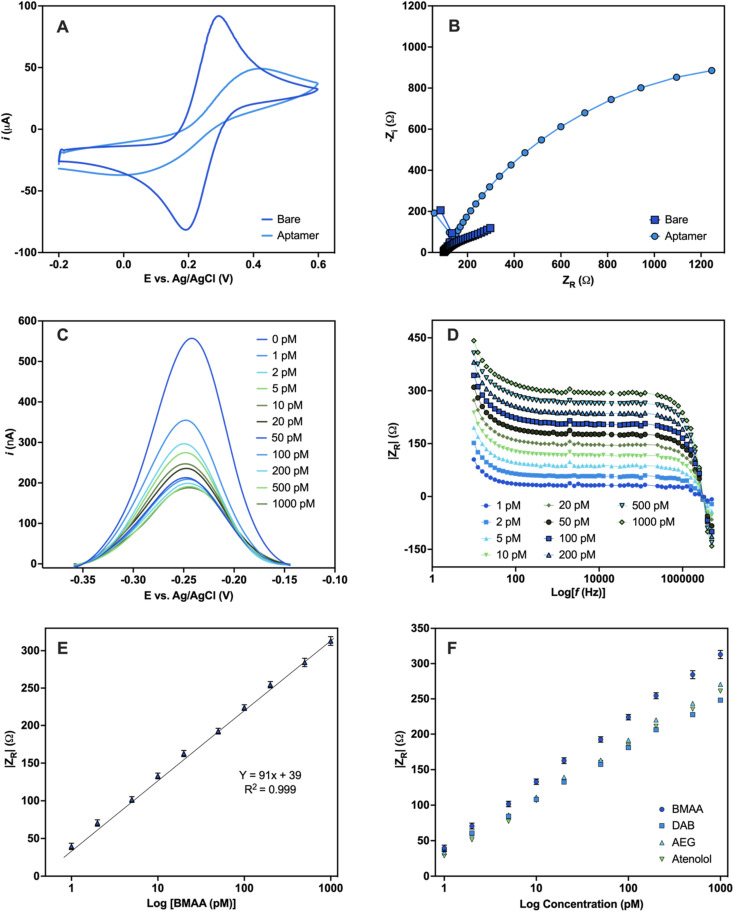
Electrochemical detection of BMAA. Characterization of Au electrodes before and after aptamer modification carried out in 5 mM [Fe(CN)_6_]^3−/4−^ redox couple solution (A) CV and (B) Nyquist plot. Electrochemical detection of the EAB sensor upon addition of varying concentrations of BMAA (0, 1, 2, 5, 10, 20, 50, 100, 200, 500, 1000 pM) performed in buffer solution (4 mM NaCl, 0.2 mM MgCl_2_, and 0.8 mM Tris at pH 7.4). (C) Changes in current measured with SWV. (D) Analytical response using the absolute value of *Z*_R_ (Ω) obtained with EIS. (E) Calibration curve of the analytical response |*Z*_R_| (Ω) against BMAA concentration (*Y* = 90.77*x* + 39, *R* = 0.999, LOD = 1.13 ± 0.03, LOQ = 1.46 ± 0.07). Measurements represent the mean of three independent experiments (*n* = 9) and the error bars represent the calculated standard error. A full analysis of the independent experiments can be found in the ESI (Fig. S9 and S10, Table S8†). (F) Comparison of the analytical response |*Z*_R_| (Ω) of BMAA and structurally related compounds (AEG, DAB, atenolol). Measurements were performed in triplicates and the error bars represent the calculated standard error. Experiments can be found in the ESI (Fig. S11†).

To assess the robustness of the aptamer, a functional validation using methods that reflect the final desired aptamer application is crucial.^47^ An electrochemical aptamer-based (EAB) sensor was developed to assess the suitability of BMAA_165_min for biosensing applications. The aptamer molecules were covalently immobilized on the surface of Au electrodes using thiol-based self-assembled monolayers (SAMs).^72^ CV and EIS were used to characterize the aptamer-modified Au electrodes in a 5 mM [Fe(CN)_6_]^3−/4−^ redox couple solution. The CV of the bare Au electrode shows the characteristic reversible redox peaks, with a peak-to-peak separation (Δ*E*) of 0.1 V (Fig. 5A). After aptamer immobilization, an increase in Δ*E* along with a decrease in the cathodic and anodic peak currents was observed. The electrostatic repulsive forces generated by the negative charges of the DNA phosphate backbone slow down the rate of electron transfer between the redox couple molecules and the Au electrode.^73^ The Nyquist plots of the aptamer-modified Au electrode shows a significant increase in resistance when compared with the bare Au electrode, which also correlates with slower electron transfer rate (Fig. 5B). The anions in the redox probe encounter more difficulty reaching the Au surface as a result of the partially occupied electrode and the repulsion produced by the negatively charged DNA.^74^ The changes observed in both the CV and EIS are consistent with the successful attachment of the aptamer on the surface of the Au electrodes.^73^

For the sensing experiments, MB was used as a conjugated redox label for its ability to change due to target-binding induced aptamer folding.^75^ MB was covalently linked to aptamers by chemical cross-linking. Additionally, the Au electrodes were blocked with MCH to minimize nonspecific adsorption and avoid the physical adsorption of the DNA aptamers on the electrode surface.^73^ The EAB sensor's reaction to varying BMAA concentrations was evaluated using SWV and EIS. With SWV, a decrease in the MB current signal with increasing BMAA concentrations was observed (Fig. 5C). This is consistent with a “signal off” system, in which the redox label moves away from the electrode upon target binding, reducing electron flow to the electrode surface.^76,77^ This occurs as a result of the target molecules obstructing the MB redox reaction or the aptamer's conformation changing upon the target.^77^ However, whether BMAA_165_min adopts a G4 structure when immobilized in the sensing platform or changes conformation during BMAA binding remains unclear.

Impedance experiments provide more information than voltammetric experiments, making them a more sensitive method of examining alterations to the electrode surface and the electrode–solution interface.^78^ EIS was recorded using the redox potential of MB (−0.2 V) at different frequencies. *Z*_R_ was used as the analytical signal to investigate the EAB sensor's response to varying concentrations of BMAA and determine its analytical range. A concentration-dependent analytical response was observed when the EAB sensor was exposed to BMAA (Fig. 5D). The impedimetric sensor achieved fast (1 min) quantitative detection of BMAA, yielding a linear response from 1 to 1000 pM (Fig. 5E). The EAB sensor showed high sensitivity and reproducibility with an LOD of 1.13 ± 0.03 pM and an LOQ of 1.46 ± 0.07 pM. It is worth noting that these values are significantly below the expected concentrations of BMAA in water sources.^79^ The EAB sensor's selectivity was tested by evaluating its cross-reactivity with the antibiotic atenolol and isomers AEG and DAB. The impedimetric EAB sensor had a higher analytical response for BMAA in comparison to the potentially interfering compounds studied, demonstrating high specificity (Fig. 5F). These findings confirmed the EAB sensor's suitability for the sensitive and selective detection of BMAA, even in the presence of closely related substances like isomers. Moreover, the overall outcomes validate the immobilized aptamer's functionality and its application in biosensing. Further studies are necessary to assess the suitability of this biosensing platform for the detection of BMAA in environmental and biological samples.

## Conclusions

The selection and characterization of the first DNA aptamers targeting cyanotoxin BMAA are presented in this work. HTS analysis revealed the enrichment of G-rich motifs during the *in vitro* selection and led to the identification of putative aptamer candidates. From the candidates that displayed BMAA binding, BMAA_159 and BMAA_165 showed the highest affinities. A comprehensive examination of these aptamers through various characterization techniques evidenced the superior specificity and selectivity of BMAA_165 (*K*_d_ = 0.32 ± 0.02 μM) and its truncated form BMAA_165_min (*K*_d_ = 0.63 ± 0.02 μM). Additionally, it was discovered that BMAA_165_min forms highly stable G4 structures. Furthermore, the development of an EAB sensor served to evaluate the suitability of BMAA_165_min in biosensing applications. Fast electrochemical detection of BMAA was achieved with high sensitivity (LOD = 1.13 ± 0.02 pM), reproducibility, and selectivity. This work establishes a framework for the creation of innovative aptamer-based analytical techniques for BMAA analysis with significant impacts on environmental and food safety monitoring.

## Author contributions

Xaimara Santiago-Maldonado: conceptualization, methodology, data analysis, writing – original draft. José A. Rodríguez-Martínez: conceptualization, resources, writing – review and editing. Luis Lopez: biosensing conceptualization, methodology, and data analysis. Lisandro Cunci: biosensing conceptualization, data analysis, resources. Marvin Bayro: NMR experiments. Eduardo Nicolau: conceptualization, writing – review and editing, resources, supervision. All authors have approved the final version of the manuscript.

## Conflicts of interest

The authors declare no competing financial interest.

## Supplementary Material

RA-014-D4RA02384F-s001
