# Supplementary material for: Selection, characterization, and biosensing applications of DNA aptamers targeting cyanotoxin BMAA
Source: RSC Adv. 2024 Apr 26;14(20):13787–800. doi: 10.1039/d4ra02384f (PMC11046380; doi:10.1039/d4ra02384f)
Supplement: RA-014-D4RA02384F-s001 [file RA-014-D4RA02384F-s001.pdf]

Electronic Supplementary Material (ESI) for RSC Advances.
This journal is © The Royal Society of Chemistry 2024

Supplementary Information

Selection, Characterization, and Biosensing Applications of DNA Aptamers Targeting Cyanotoxin BMAA

Xaimara Santiago-Maldonado[1], José A. Rodríguez-Martínez[2], Luis López[1], Lisandro Cunci[1], Marvin Bayro[1,3], Eduardo Nicolau[1,3*]

[1]Department of Chemistry, University of Puerto Rico, San Juan, PR 00925-2437, United States

[2]Department of Biology, University of Puerto Rico, San Juan, PR 00925-2437, United States

[3]Molecular Science Research Center, University of Puerto Rico, San Juan, 00931-3346, United States

Corresponding Author*

Phone: 787-292-9820, fax: 787-522-2150; email: eduardo.nicolau@upr.edu

Table of Contents

Figure S1. Chemical structure BMAA

Figure S2. Chemical structure of the counter-targets

Table S1. DNA oligos used for SELEX

Table S2**.** Sequences of the DNA aptamers

Table S3. Beads-SELEX scheme for selection of BMAA-specific aptamers

Table S4. Identification of overrepresented motifs using AptaTRACE

Table S5. Apparent K_d values of the initial screening

Figure S3. Screening for binders using the SG fluorescence displacement assay.

Figure S4. Secondary structure of the aptamers with the highest affinity

Figure S5. Non-specific binding BMAA-conjugated fluorescence assay

Figure S6. Amino proton region of ^{1}H NMR spectra

Figure S7. Replicates SG fluorescence displacement assay

Table S6. Results of the non-linear fit SG fluorescence displacement assay

Figure S8. Replicates BMAA-conjugated fluorescence assay

Table S7. Results of the non-linear fit BMAA-conjugated fluorescence assay

Figure S9. Independent experiments for the electrochemical detection of BMAA

Figure S10. Calibration curves of the EAB sensor

Table S8. Determination of LOD and LOQ values for the EAB sensor

Figure S11. Electrochemical detection of AEG, atenolol, and DAB

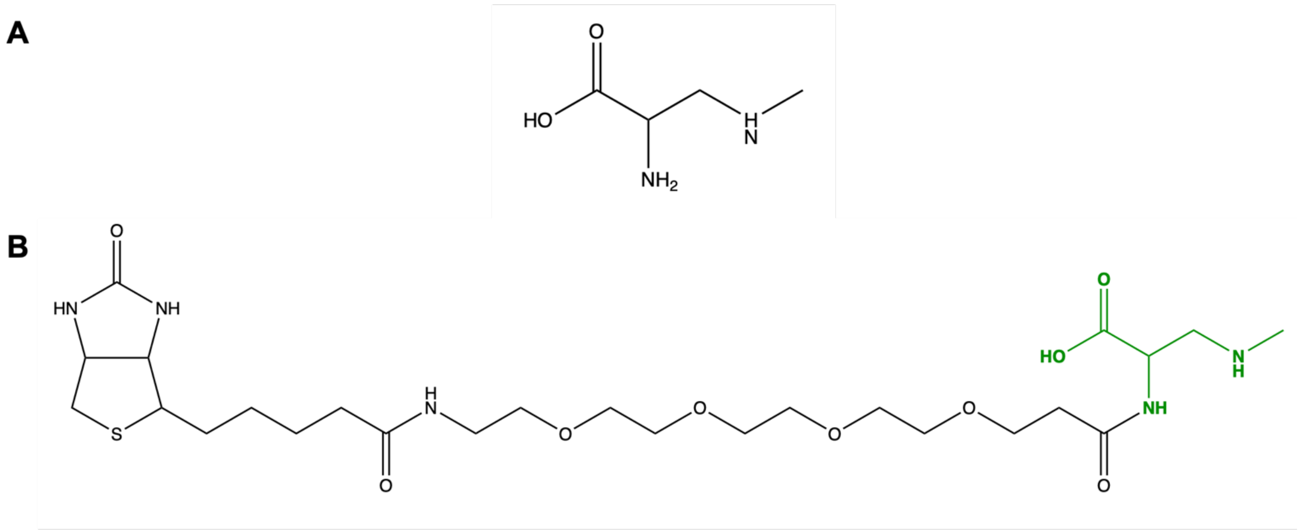

Figure S1. Chemical structures A) BMAA B) Biotinylated BMAA consisting of a biotin moiety with a PEG_4 spacer arm covalently attached to the primary amine of BMAA (shown in green).

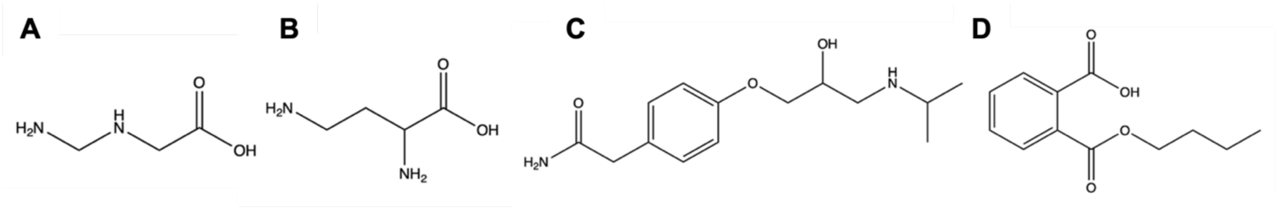

Figure S2. Chemical structures of counter targets, including isomers A) aminoethylglycine (AEG) B) 2,4-diaminobutyric acid (DAB) and other low molecular-weight emerging contaminants C) Atenolol D) Mono-butyl phthalate.

Table S1. DNA oligos used for SELEX

**Name**	**Sequence (5'- 3')**
Library	TGTACCGTCTGAGCGATTCGTAC (N_{34}) AGCCAGTCA GTGTTAAGGAGTGC
Forward primer	TGTACCGTCTGAGCGATTCGTAC
Forward primer + biotin	/Biot/ GCA CTC CTT AAC ACT GAC TGG CT
Reverse primer	GCACTCCTTAACACTGACTGGCT
xGen full-length UDI-UMI adapter index i7	GATCGGAAGAGCACACGTCTGAACTCCAGTCAC $[i7]_8$ UMI ATCTCGTATGCCGTCTTCTGCTTG
xGen full-length UDI-UMI adapter index i5	AATGATACGGCGACCACCGAGATCTACAC $[i5]_8$ ACACTCTTTCCCTACACGACGCTCTTCCGATCT

Table S2. Sequences of the DNA aptamers. Overrepresented motifs are underlined.

**Full-length**	**N_{34} Sequence (5’- 3’)**
BMAA_159	GGCTCGGCCCTGGGTGAGGGGCGCAGTGGGGTGT
BMAA_165	GGGCGTGTGTGGGAGGGGGCACTTCGTCGGGGTG
BMAA_172	GGGGGCCAGTGAATGAGTTGGGGTGGTAATGGGG
BMAA_96	AGAGGGGGTGGCGGGTGTCTGGAGTGGAGGGTGG
BMAA_38	GGGGGTTCACGCTCGAGGGGGCCACAGGGGGAGT
BMAA_165_scrambled	AGTCGTTGGGGGGGGGGTGAGCGTTGCGCGCGTAGCC
**Truncated**	**Sequence (5’- 3’)**
BMAA_159_min	GGCTCGGCCCTGGGTGAGGGGCGCAGTGGGGTGTAGCCAG
BMAA_165_min	GGGCGTGTGTGGGAGGGGGCACTTCGTCGGGGTGAGCCAG
**Biosensing**	
BMAA_165_min	/5ThioMC6-D/GGGCGTGTGTGGGAGGGGGCACTTCGTCGGGGTG/3AmMO/-3’

Table S3. Beads-SELEX scheme for selection of BMAA-specific aptamers (IT = immobilization target, IS = immobilization substrate, CE = competitive elution, CTs = counter targets, IM = isomers).

**Round**	**Positive Selection**	**Time**	**Round**	**Negative Selection**	**Time**
**1**	IT	48 h		-	-
**2**	IT	24 h		-	-
**3**	IT	18 h	4	IS	1 h
**5**	IT	12 h	6	IS	3 h
**7**	IT	6 h	8	IS	6 h
**9**	IT / CE 500 μM BMAA	3 h/1 h	10	IT / 1 μM CTs	3 h / 1.5 h / 1.5 h
**11**	IT / CE 500 μM BMAA	1 h / 30 min	12	IT / 1 μM IM AEG	3 h /3 h
**13**	IT / CE 100 μM BMAA	30 min / 15 min	14	IT / 1 μM IM L-DAB	3 h / 3 h
**15**	IT / CE 10 μM BMAA	15 min / 5 min	16	IT / 1 μM IMs (serial)	1 h / 1 h / 1 h
**17**	IT / CE 1 μM BMAA	5 min / 2 min		-	-
**18**	IT / CE 100 nM BMAA	1 min / 1 min		-	-

Table S4. Identification of overrepresented motifs using AptaTRACE

Selection Method	Motif Profile	Seed	Seed P-value	Seed frequency	Motif frequency
**Beads-SELEX**	GGGGG	TGGGGG	0.050	3.93%	8.29%
	GGGAG	GGGGAG	0.045	2.87%	4.44%
	GAGGGG	GAGGGG	0.031	2.69%	2.69%
	GGAGGG	GGAGGG	0.029	2.54%	2.54%

Table S5. Apparent K_d values of the initial screening were obtained using the SG fluorescence displacement assay. The K_d values were determined through non-linear regression analysis by fitting the data with one site-specific binding equation using GraphPad Prism 10.2.0. No binding refers to the absence of a concentration-dependent trend within the studied range of concentrations.

ID	K_d (95% CI)	K_d (μM)
BMAA_159	1.02 – 8.66	3.03
BMAA_165	0.149 – 0.690	0.234
BMAA_172	5.23 – 9.56	6.50
BMAA_96	No binding*	-
BMAA_38	3.08 - 102	18.6

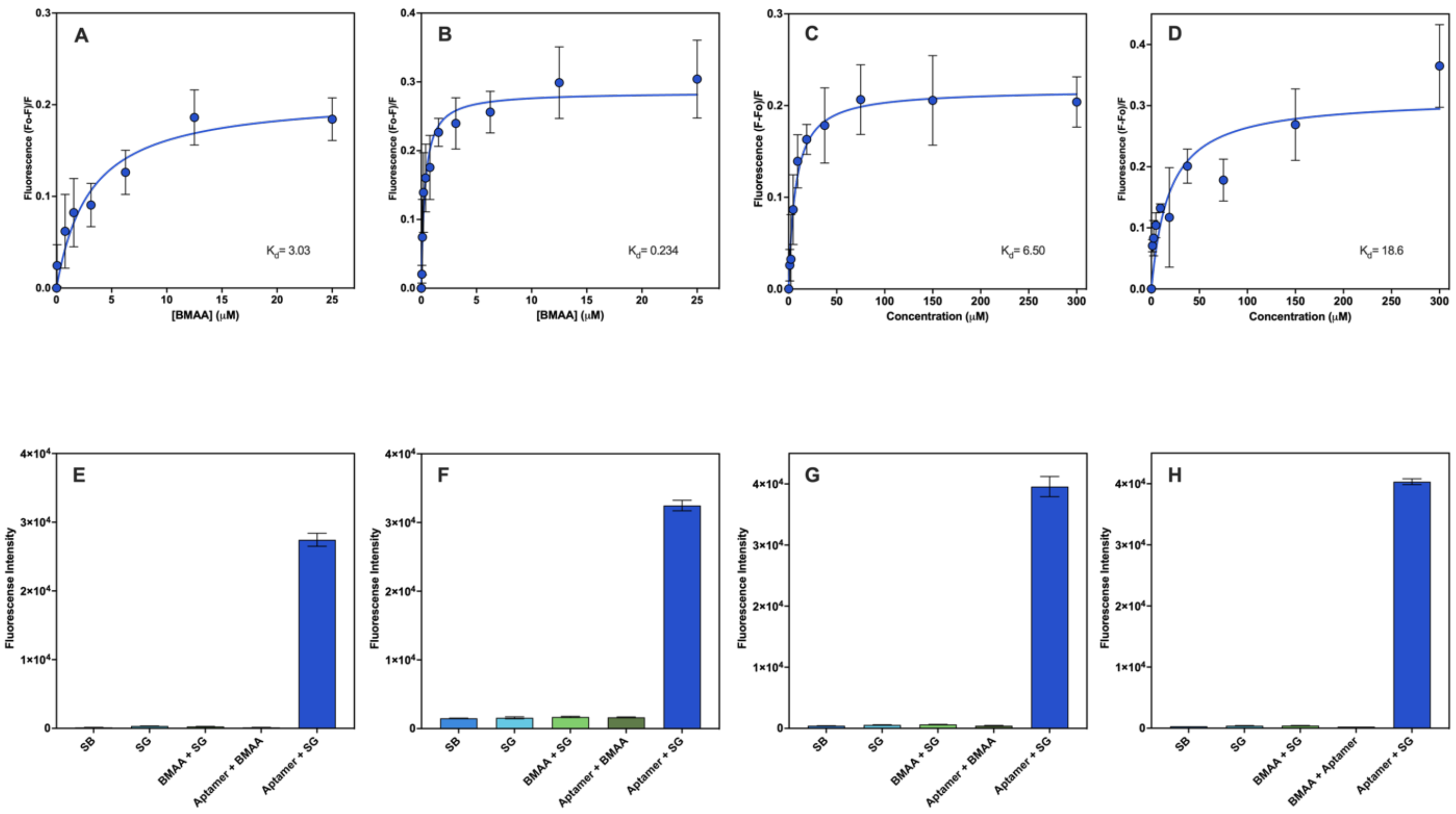

Figure S3. Screening for binders using the SG fluorescence displacement assay. Binding isotherms of A) BMAA_159 (K_d = 3.03) B) BMAA_165 (K_d = 0.234) C) BMAA_172 (K_d = 6.50) D) BMAA_38 (K_d = 18.6). BMAA_96 did not show a concentration-dependent trend within the studied range of concentrations. The K_d values were determined through non-linear regression analysis by fitting the data with one site-specific binding equation using GraphPad Prism 10.2.0. Control samples of E) BMAA_159 F) BMAA_165 G) BMAA_172 H) BMAA_38. Measurements were performed in triplicates and the error bars represent the calculated standard error.

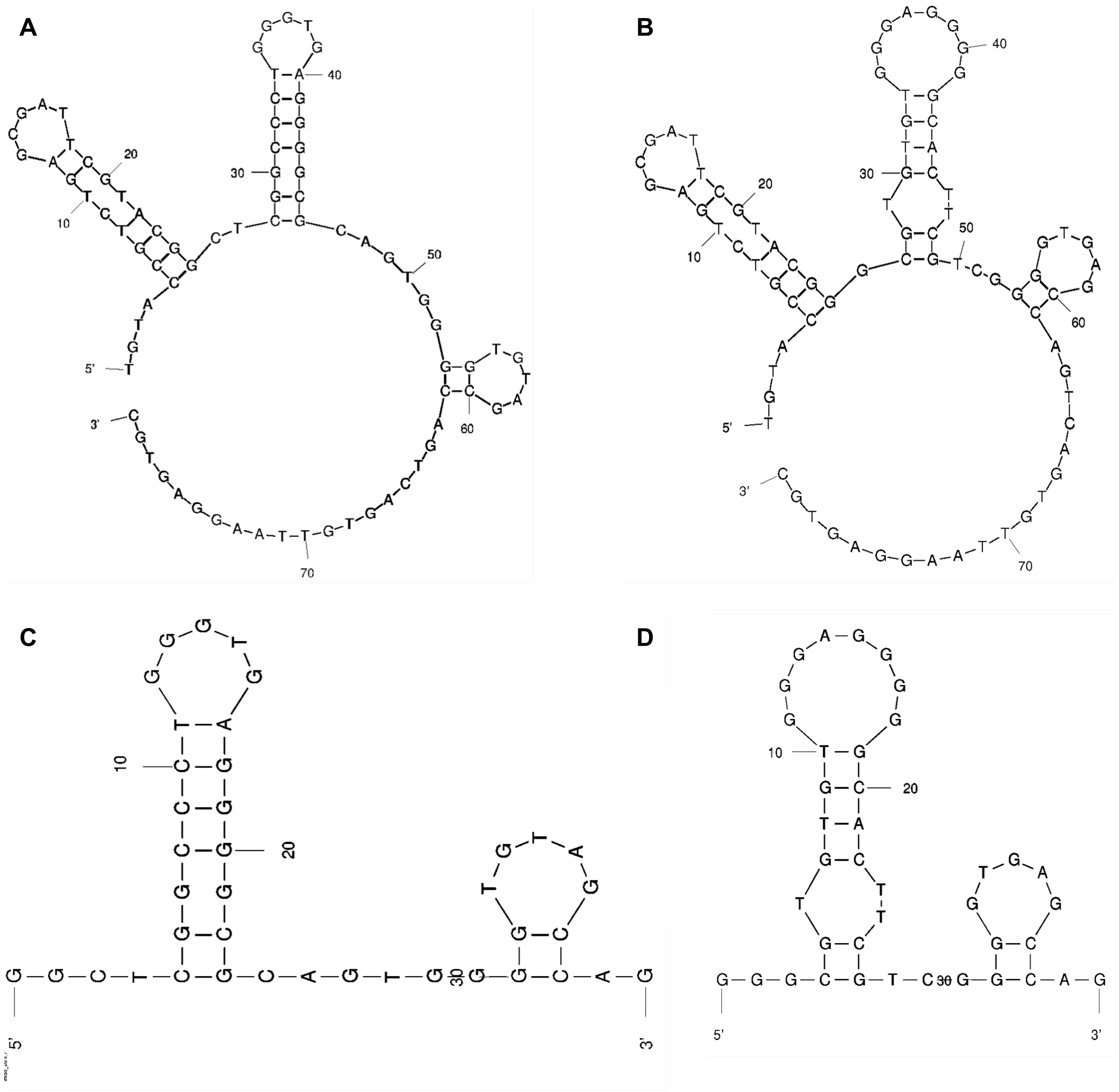

Figure S4. Secondary structure of the aptamers with the highest affinity A) BMAA 159 B) BMAA_165 C) BMAA_159_min and D) BMAA_165_min. Structures were predicted using the Mfold software considering ambient temperature and ionic conditions of the selection buffer (100 mM NaCl and 2 mM $MgCl_2$).

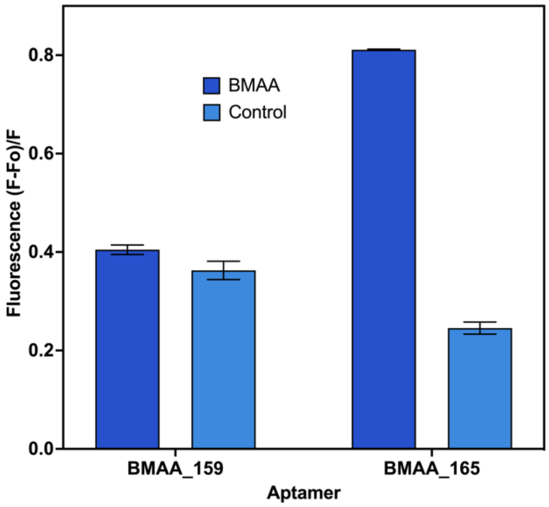

Figure S5. Non-specific binding of truncated aptamers measured with the BMAA-conjugated fluorescence assay. Normalized fluorescence response of the aptamers (1 μM) with BMAA and without (control). Measurements were performed in triplicates and the error bars represent the calculated standard error.

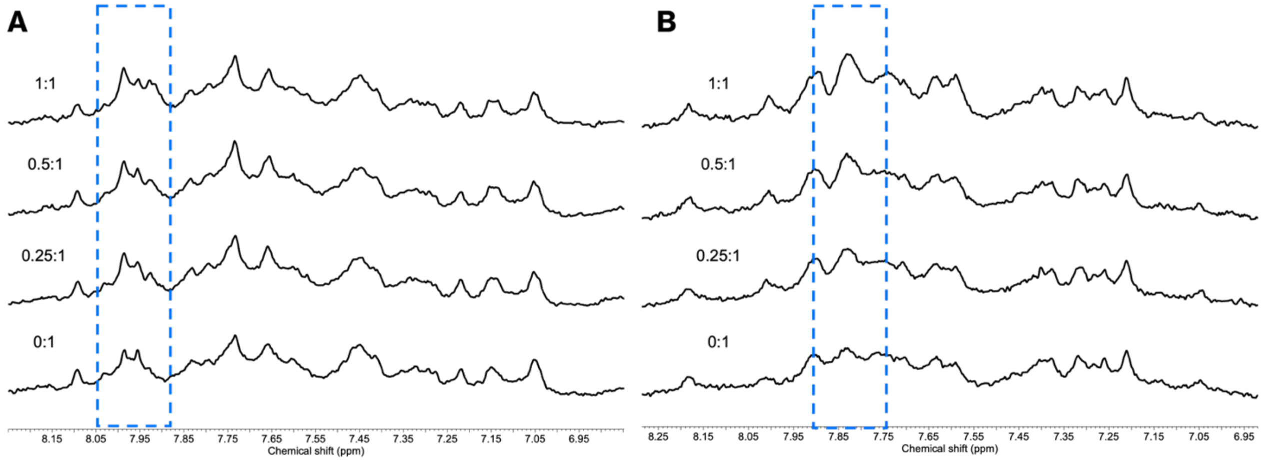

Figure S6. Amino proton region of the ^{1}H NMR spectra recorded in 20 mM sodium phosphate buffer with 100 mM NaCl and 2 mM $MgCl_2$ at 700 MHz ^{1}H frequency of A) BMAA_159_min B) BMAA_165_min. Aptamer solutions (20 μM) were incubated with increasing molar concentrations of BMAA. Chemical shift perturbations are highlighted, indicating a conformational change in the aptamer upon binding.

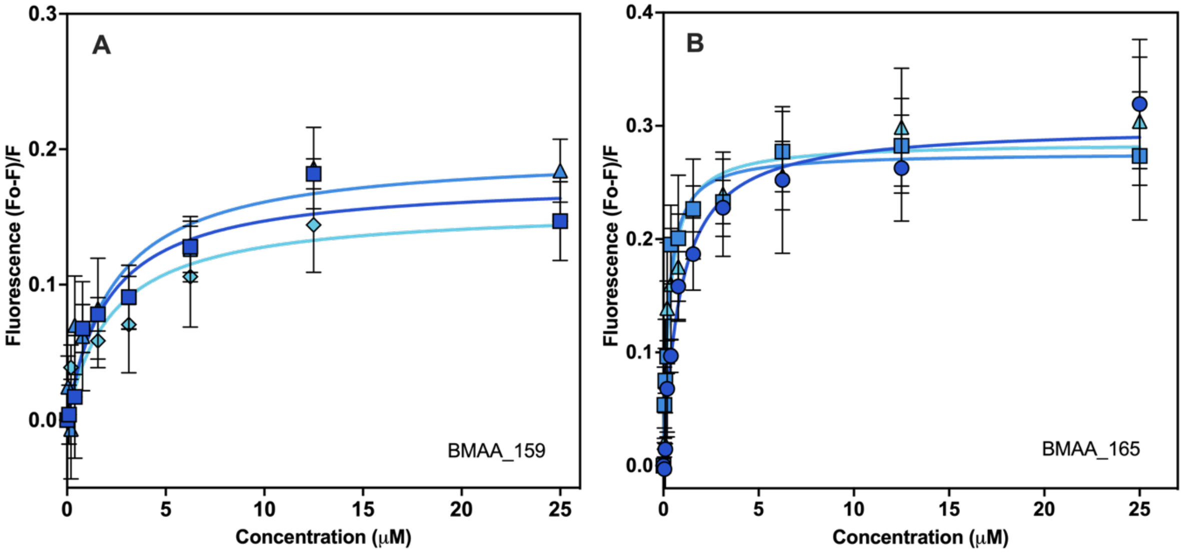

Figure S7. Independent experiments SG fluorescence displacement assay. Binding isotherms were obtained using GraphPad Prism 10.2.0 for A) BMAA_159 and B) BMAA_165. Measurements were performed in triplicates and the error bars represent the calculated standard error.

Table S6. Apparent K_d values were obtained using the SG fluorescence displacement assay. Each measurement represents an independent experiment. The K_d values were determined through non-linear regression analysis by fitting the data with one site-specific binding equation using GraphPad Prism 10.2.0.

**Aptamer**	**K_d (95% CI)**	**K_d (μM) Best fit**	**R^2**	**K_d (μM) Mean**	**Standard Error**
BMAA_159	0.93 - 4.38	2.06	0.94	2.2	0.1
	0.78 – 6.50	2.30	0.90		
	0.84 – 5.91	2.27	0.92		
BMAA_165	0.60 - 1.24	0.86	0.98	0.32	0.02
	0.18 – 0.37	0.26	0.98		
	0.20 – 0.51	0.32	0.97		

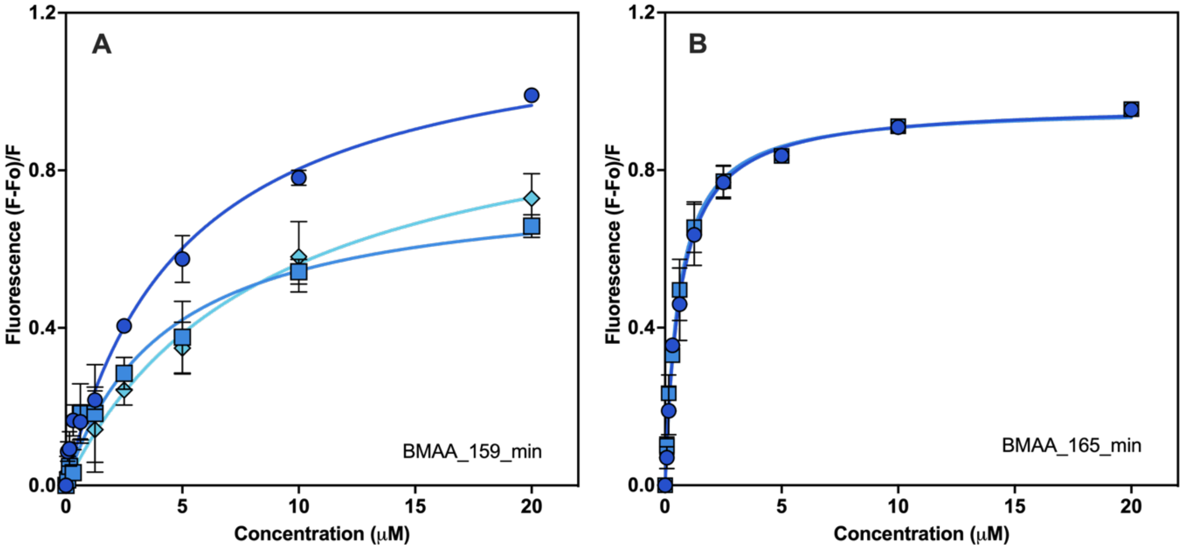

Figure S8. Independent experiments BMAA-conjugated fluorescence assay. Binding isotherms were obtained using GraphPad Prism 10.2.0 for A) BMAA_159 B) BMAA_165. Measurements were performed in triplicates and the error bars represent the calculated standard error.

Table S7. Apparent K_d values were obtained using the BMAA-conjugated fluorescence assay. Each measurement represents an independent experiment. The K_d values were determined through non-linear regression analysis by fitting the data with one site-specific binding equation using GraphPad Prism 10.2.0.

Aptamer	K_d (95% CI)	K_d (μM) Best fit	R^2	K_d (μM) Mean	Standard Error
BMAA_159_min	3.12 – 8.26	5.02	0.98	6	1
	2.55 – 7.03	4.20	0.98		
	4.53 – 17.72	8.54	0.97		
BMAA_165_min	0.56 – 0.75	0.65	0.99	0.63	0.02
	0.52 – 0.66	0.58	0.99		
	0.55 – 0.76	0.65	0.99		

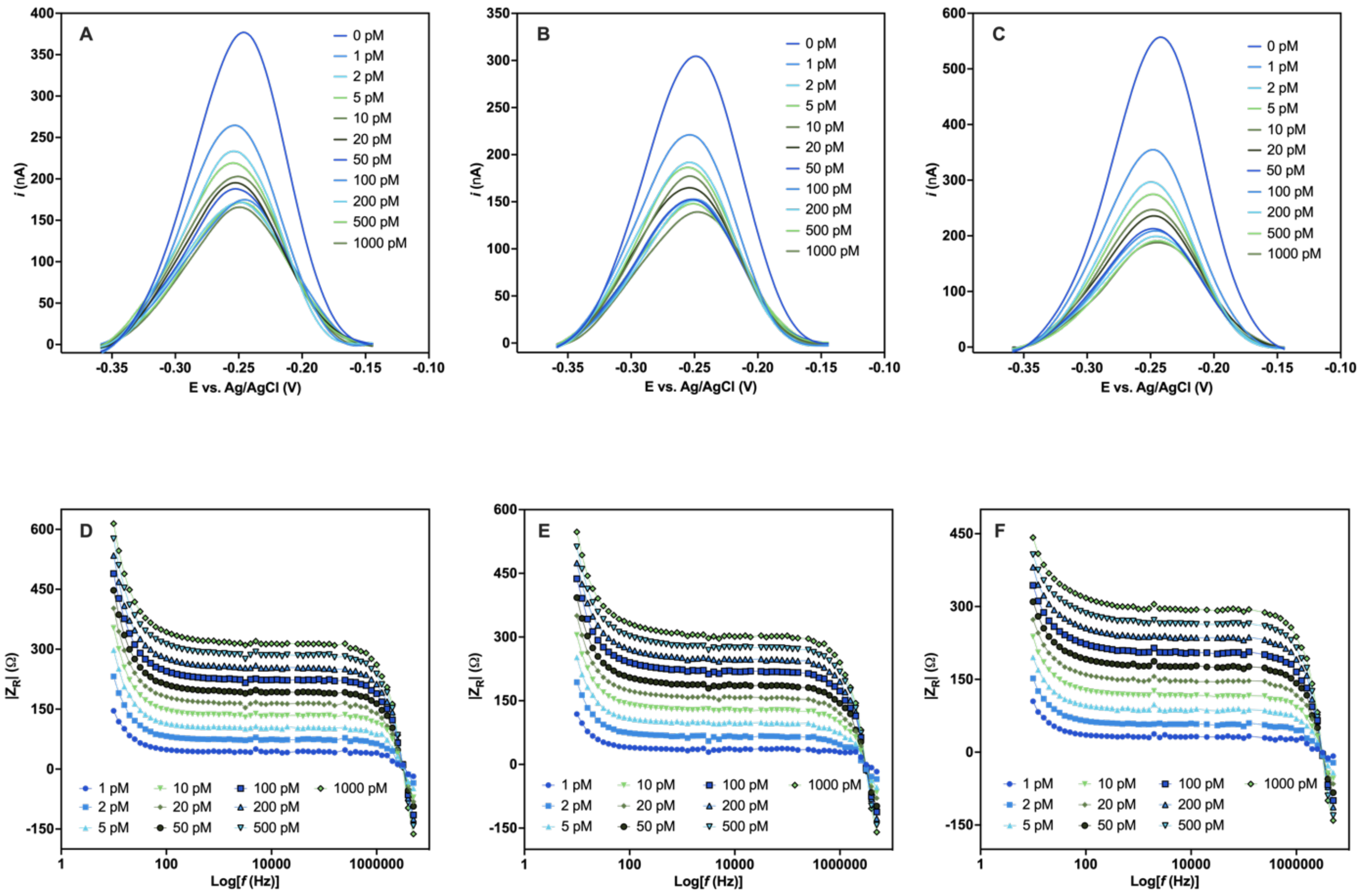

Figure S9. Independent experiments for the electrochemical detection of the EAB sensor upon addition of varying concentrations of BMAA (0, 1, 2, 5, 10, 20, 50, 100, 200, 500, 1000 pM) performed in buffer solution (4 mM NaCl, 0.2 mM $MgCl_2$, and 0.8 mM Tris at pH 7.4). Changes in current measured using SWV A) Electrode 1 B) Electrode 2 C) Electrode 3. Analytical response using the absolute value of Z_R (Ω) obtained using EIS D) Electrode 1 E) Electrode 2 F) Electrode 3. Measurements were performed in triplicates.

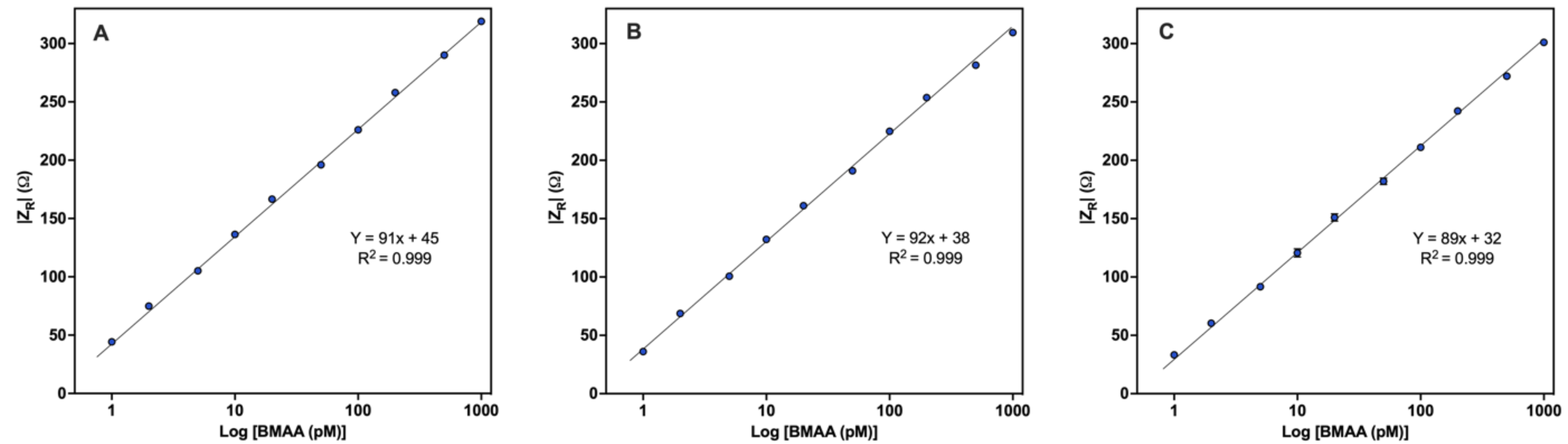

Figure S10. Independent experiments of the EAB sensor. Calibration curves of the analytical response $|Z_R|$ (Ω) against BMAA concentration A) Electrode 1 (Y = 91x + 45, R= 0.999, n=3) B) Electrode 2 (Y = 92x + 38, R= 0.999, n=3) C) Electrode 3 (Y = 89x + 32, R= 0.999, n=3). Simple linear regression analysis was obtained using GraphPad Prism 10.2.0.

Table S8. Determination of LOD and LOQ values for the EAB sensor. Simple linear regression analysis was obtained using GraphPad Prism 10.2.0. LOD = 3σ/S and LOQ = 10σ/S, where S is the slope of the curve and σ is the standard deviation. SE represents the calculated standard error.

Electrode	S	σ	LOD (pM)	Mean LOD (pM)	SE	LOQ (pM)	Mean LOQ (pM)	SE
1	91	1	1.11			1.38		
2	92	2	1.17	1.13	0.03	1.62	1.46	0.07
3	89	1	1.11			1.38		

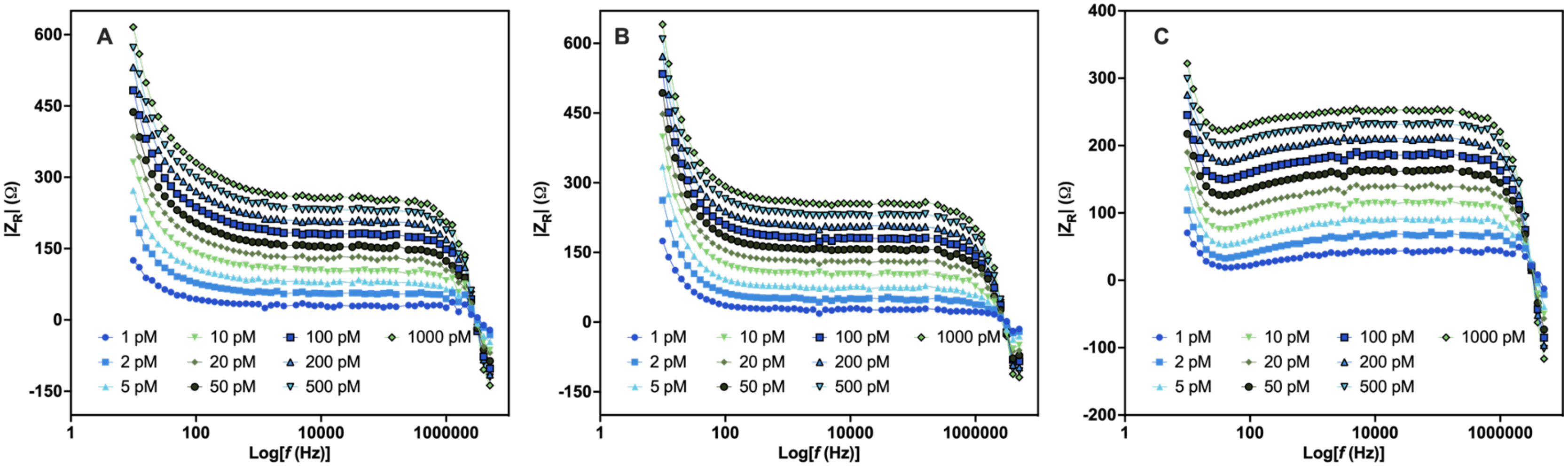

Figure S11. Electrochemical detection of the EAB sensor upon addition of varying concentrations of analyte (0, 1, 2, 5, 10, 20, 50, 100, 200, 500, 1000 pM) performed in buffer solution (4 mM NaCl, 0.2 mM $MgCl_2$**,** and 0.8 mM Tris at pH 7.4). Analytical response using the absolute value of Z_R (Ω) obtained using EIS of analyte A) AEG B) Atenolol 2 C) DAB. Measurements were performed in triplicates.
